# Transverse Sinus Stenosis in Venous Pulsatile Tinnitus Patients May Lead to Brain Perfusion and White Matter Changes

**DOI:** 10.3389/fnins.2021.732113

**Published:** 2021-12-08

**Authors:** Xiaoshuai Li, Ning Xu, Xuxu Meng, Chihang Dai, Xiaoyu Qiu, Heyu Ding, Han Lv, Rong Zeng, Jing Xie, Pengfei Zhao, Zhenghan Yang, Shusheng Gong, Zhenchang Wang

**Affiliations:** ^1^Department of Radiology, Beijing Friendship Hospital, Capital Medical University, Beijing, China; ^2^Department of Otolaryngology Head and Neck Surgery, Beijing Friendship Hospital, Capital Medical University, Beijing, China

**Keywords:** transverse sinus stenosis, pulsatile tinnitus, brain perfusion, white matter, arterial spin labeling

## Abstract

**Objective:** Transverse sinus stenosis (TSS) is associated with various symptoms, but whether it can lead to pathological brain changes is unclear. This study aimed to investigate brain changes in venous pulsatile tinnitus (PT) patients with TSS.

**Materials and Methods:** In this study, fifty-five consecutive venous PT patients and fifty age- and gender-matched healthy controls (HCs) were investigated. In CT venography, the combined conduit score (CCS) was used to assess the degree of TSS in venous PT patients. Magnetic resonance venography was used to assess TSS in HCs. All the participants had undergone arterial spin labeling and structural MRI scans.

**Results:** Two patients without TSS and ten HCs with TSS were excluded. Fifty-three venous PT patients with TSS and 40 HCs without TSS were included in this study. All the patients had unilateral cases: 16 on the left and 37 on the right. Based on the CCS, the patients were divided into high-degree TSS (a score of 1–2) (*n* = 30) and low-degree TSS groups (a score of 3–4) (*n* = 23). In the whole brain and gray matter, the patients with high-degree TSS showed decreased cerebral blood flow (CBF) compared with patients with low-degree TSS as well as HCs (*P* < 0.05), and no significant difference in CBF was found in patients with low-degree TSS and HCs (*P* > 0.05). In white matter (WM) regions, the patients with high-degree TSS exhibited decreased CBF relative to the HCs (*P* < 0.05). The incidence of cloud-like WM hyperintensity was significantly higher in the above two patient groups than in the HC group (*P* < 0.05).

**Conclusion:** TSS in venous PT patients may lead to decreased CBF and cloud-like WM hyperintensity. These neuroimaging findings may provide new insights into pathological TSS in venous PT.

## Introduction

Pulsatile tinnitus (PT) is an auditory perception synchronized with vascular pulsation (Wang et al., 2014). According to the blood vessels of origin, PT is divided into the arterial, venous and arteriovenous types (Hofmann et al., 2013), of which venous PT accounts for 84% of the PT population (Lyu et al., 2018). The sound originates from turbulent flow in the transverse-sigmoid sinus, which is then perceived by the inner ear (Bae et al., 2015). Transverse sinus stenosis (TSS) is one of the most common anomalies in venous PT, and it is also a clear etiology of this condition. A previous study reported that 84.6% of venous PT patients have varying degrees of bilateral TSS (Hewes et al., 2020). After stent placement, PT can disappear completely (Baomin et al., 2014). With the rapid development of imaging techniques, an increasing number of studies have focused on the relationship between TSS and the central nervous system (Saindane et al., 2014; Morris et al., 2017; Favoni et al., 2019).

The intracranial venous system is a complex three-dimensional structure with greater anatomic variation and asymmetry than the arterial structure (Scott and Farb, 2003). Additionally, the former is susceptible to physiological factors such as posture, breathing, and the hydration status (Werner et al., 2011). Some additional factors, such as enlarged arachnoid granules, septa, and congenital causes, can lead to TSS (Durst et al., 2016), increasing the susceptibility to this venous pathology.

Currently, TSS is the cause of the pathological conditions of venous PT (Baomin et al., 2014) and idiopathic intracranial hypertension (Farb et al., 2003; Carvalho et al., 2017). However, few studies have focused on the neuroimaging characteristics of symptomatic TSS. A previous study used semiquantitative single-photon emission computed tomography (SPECT) to identify decreased brain perfusion and cloud-like white matter (WM) hyperintensity in symptomatic internal jugular vein (IJV) stenosis patients (Zhou et al., 2019). Bai et al. (2019) further found that patients with symptomatic TSS also have cloud-like WM hyperintensity. Ding et al. reported a PT patient with TSS and decreased brain perfusion; after TSS was resolved by stent placement, the hypoperfusion state returned to normal (Ding et al., 2019). These findings indicate that TSS may cause pathological changes in the central nervous system. However, the above studies assessed brain perfusion using SPECT (Ding et al., 2019; Zhou et al., 2019), which is a semiquantitative and invasive technique. Arterial spin labeling (ASL) uses magnetically labeled arterial blood water as an endogenous tracer, which can non-invasively and quantitatively assess brain perfusion (Ha et al., 2019). Additionally, because stent placement requires the long-term use of anticoagulants, sigmoid sinus wall reconstruction is the main surgical treatment for venous PT in clinical practice (Zhao et al., 2016; Eisenman et al., 2018; Hewes et al., 2020) but does not resolve TSS. In the present study, we aimed to quantitatively evaluate brain perfusion changes by ASL and investigate white matter changes in venous PT patients with TSS, possibly helping clinicians better understand such abnormalities and choose treatment options.

## Materials and Methods

### Subjects

This study protocol was approved by local institutional review boards. All the participants offered written informed consent.

Fifty-five consecutive venous PT patients from the Department of Otolaryngology Head and Neck surgery were included in the study between January 2018 and December 2020. All the patients showed pulse-synchronous tinnitus, which disappeared after compression of the ipsilateral internal jugular vein. The age was between 18 and 50 years. These patients had no neurological disorders, such as headache, papilledema, decreased vision, sleep disturbance or memory deterioration. The audiometric and otoscopic examination results were normal. CT arteriography/venography (CTA/V), digital substraction angiography (DSA), and ASL were performed in each patient before treatment. The exclusion criteria for venous PT patients were as follows: (1) systemic diseases such as hypertension, hyperlipidemia, diabetes, and kidney disease; (2) carotid or intracranial artery abnormalities, including congenital malformation, large or small vascular disease and stenosis; (3) IJV stenosis or thrombosis; (4) significant stenosis or hypoplasia of venous sinuses in HCs; (5) neurodegenerative diseases or neuroinflammation; (6) psychiatric or neurological disorders; (7) history of head trauma or tumor; (8) drug and alcohol abuse in the last 3 months; and (9) contraindications for MRI. The Tinnitus Handicap Inventory (THI) score was used to assess the severity of PT.

Fifty age- and gender-matched healthy controls (HCs) were enrolled. The exclusion criteria for HCs were the same as those for venous PT patients above.

### Imaging Technique

MR arteriography/venography (MRA/V) of all the participants was performed using a Philips 3.0T MRI unit (Ingenia; Philips Healthcare, Best, Netherlands). The parameters of 3D time of flight (TOF) MRA were as follows: repetition time (TR), 19 ms; echo time (TE), 3.5 ms; flip angle (FA), 18°; field of view (FOV), 200 × 200 mm; matrix, 400 × 256; slice thickness, 1.1 mm, no gap; 156 slices. The parameters of 3D phase-contrast MRV were as follows: TR, 17 ms; TE, 6.2 ms; FA, 10°; FOV, 173 × 173 × 192 mm; velocity encoding, 15 cm/s; matrix, 144 × 108 × 120.

All venous PT patients had undergone CTA/V examination using a 64-slice CT system (Philips, Best, Netherlands) with a bolus tracking program. The parameters for CTA/V were as follows: 300 mAs; 100 kV; collimation, 64 × 0.625 mm; matrix, 512 × 512; and rotation time, 0.75 s; contrast agent (iopamidol, 370 mg/ml iodine; Bracco, Shanghai, China): 1.5 ml/kg, 5 ml/s. Images were reconstructed on a postprocessing workstation with the following parameters: thickness, 1 mm, no gap; standard algorithms (width 700 Hu; level 200 Hu) for soft tissue reconstruction; bone algorithms (width 4,000 Hu; level 700 Hu) for bone reconstruction.

**FIGURE 1:**
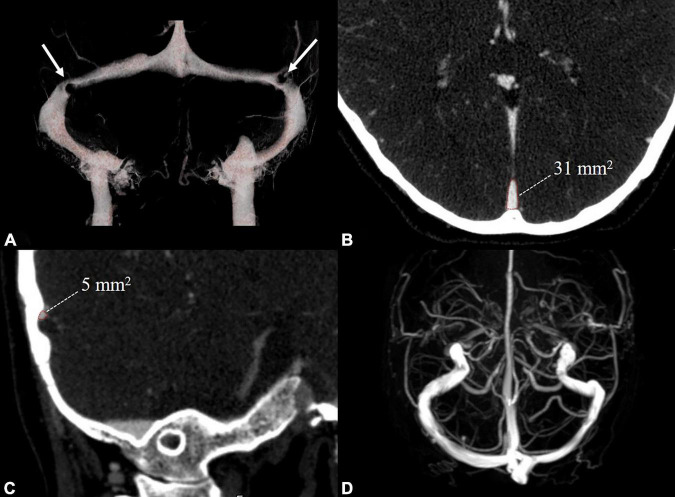
Examples of radiological evaluation in a venous PT patient with TSS and a healthy control. CTV **(A)** showed bilateral TSS in the venous PT patient (white arrow). The cross-sectional area of the distal superior sagittal sinus **(B)** and TSS **(C)** were measured perpendicular to the venous sinus. MRV **(D)** showed no TSS in the healthy control. TSS: transverse sinus stenosis.

Head MRA/V and cervical Doppler ultrasound were used to exclude intracranial and cervical arteriovenous abnormalities in HCs. In venous PT patients, head CTA/V and DSA were used to assess intracranial and cervical arteries and veins. CTV was used to assess the severity of TSS in venous PT patients. Based on the improvement of the method proposed by Farb et al. (2003), the TSS in venous PT patients was assessed by dividing the cross-sectional area of the most stenotic segment of the transverse sinus by that of the distal superior sagittal sinus (Figure 1). The left and right transverse sinuses were independently divided into 0–4 grades as follows: grade 0, discontinuity (gap); grade 1, hypoplasia or severe stenosis (<25%); grade 2, moderate stenosis (25–50%); grade 3, mild stenosis (50–75%); and grade 4, normal (>75%). This method calculates the combined conduit score (CCS) as the sum of right and left scores, which can be used to reflect the overall venous drainage. Normally, the left and right transverse sinuses would reveal a score of 4, and the final CCS would show a score of 8. A transverse sinus is characterized as dominant if its largest cross-sectional area is greater than 150% of that of the smaller side (Li et al., 2021).

All the participants had undergone brain structure and perfusion scans using a GE 3.0T MRI unit (Discovery MR750W; GE, Milwaukee, WI, United States) using an eight-channel phased-array head coil. The structural images included a 3D T1-brain volume (T1-BRAVO) sequence [TR/TE/inversion time (TI), 8.4/3.2/450 ms; slice thickness, 1 mm without gap; FA, 12°; 196 slices], T2-weighted imaging (T2WI: TR/TE, 5,637/130 ms; slice thickness, 6 mm; gap: 7 mm; 20 slices), T2 fluid attenuated inversion recovery (T2 FLAIR: TR/TE/TI, 8,400/88/2,404 ms; slice thickness, 6 mm; gap, 7 mm; FA, 160°; 20 slices) and diffusion-weighted imaging (DWI: TR/TE, 4,880/79 ms; slice thickness, 6 mm; gap, 7 mm; b value: 0, 1,000 s/mm^2^; FA, 90°; 20 slices). The perfusion images were acquired using a 3D pseudocontinuous ASL sequence with fast spin-echo acquisition (TR/TE, 4,854/10.7 ms; slice thickness, 4 mm, no gap; number of excitations, 3; postlabel delay, 2,025 ms; FOV, 240 mm × 240 mm; in-plane resolution, 3.37 mm × 3.37 mm; FA, 111°; 36 slices). During the imaging process, the participants were instructed to close their eyes, move as little as possible, stay awake and think of nothing. Earplugs were provided to reduce noise.

### Cerebral Blood Flow Calculation

Using a single compartment model, the ASL difference images were calculated after subtracting the label images from the control images. Next, we obtained the cerebral blood flow (CBF) maps from the ASL difference images. The preprocessing of the CBF images was as follows. Statistical Parametric Mapping (SPM8) software was used to normalize the CBF maps to the Montreal Neurological Institute (MNI) space (Zhuo et al., 2017; Guo et al., 2018). Specifically, the CBF images of all the HCs were coregistered to a PET perfusion template in the MNI space to obtain a standard CBF template. We coregistered the CBF images of all HCs and PT patients to this standard CBF template and resampled the data to 2 × 2 × 2 mm. In the data processing and analysis of brain imaging (DPABI) software package, the CBF values (ml/100 g/min) of the gray matter (GM), WM and whole brain in each participant were extracted using specific GM, WM and whole brain masks, respectively.

### White Matter Change Evaluation

All the participants had undergone brain structure scans. Based on previous studies (Bai et al., 2019; Zhou et al., 2019), the WM change was defined as bilaterally symmetrical cloud-like hyperintensity in the bilateral centrum semiovale and/or periventricular area on the T2 FLAIR sequence and was evaluated by three neuroradiologists (with 6, 8, and 12 years of experience) blinded to the clinical data. Inconsistent cases reached a consensus through consultation.

### Statistical Analysis

Statistical analysis was performed using SPSS software, version 22.0 (IBM, Chicago, IL). Fisher’s exact test and two-sample *t*-test were used to explore the differences in the clinical and demographic data between the venous PT patients and HCs. To compare CBF among the three groups, one-way analysis of variance (Bonferroni correction) was used. Chi-squared test was used to compare the difference in the incidence of cloud-like WM hyperintensity among the three groups (Bonferroni correction).

## Results

### Demographic and Clinical Characteristics

Two venous PT patients without TSS and ten HCs with TSS were excluded. Fifty-three patients and 40 HCs were enrolled in this study. The prevalence of TSS was 96.4% in venous PT patients and 20% in HCs. All the patients had unilateral PT, 16 cases on the left and 37 cases on the right. The mean PT duration was 28.0 ± 23.8 months, and the mean THI score was 50.8 ± 22.2. The patients and HCs were well matched for age (two-sample *t*-test, *P* = 0.100), gender (Fisher’s exact test, *P* = 1.000) and body mass index (two-sample *t*-test, *P* = 0.416) (Table 1).

**TABLE 1:** Characteristics of the venous PT patients and HCs.

	PT (*n* = 53)		HC (*n* = 40)	*P*
Age (year)	35.5 ± 7.0		37.9 ± 6.7	0.100^a^
Gender (male/female)	5/48		3/37	1.000^b^
BMI	23.0 ± 2.5		22.6 ± 2.0	0.416^a^
Side (left/right)	16/37			
PT duration (months)	28.0 ± 23.8			
THI score	50.8 ± 22.2			
TSS degree	High-degree	Low-degree		
CCS	1–2 score	3–4 score		
Number	30	23		

*The data are presented as mean ± standard deviation.*

*PT, pulsatile tinnitus; HC, healthy control; BMI, body mass index; THI, Tinnitus Handicap Inventory; CCS, combined conduit score; TSS, transverse sinus stenosis.*

*^a^Two-sample t-test.*

*^b^Fisher’s exact test.*

### Radiological Evaluation

No obvious abnormalities were found in the intracranial arteries and cervical arteriovenous systems of all the participants. The dominance of the transverse sinus in 44 patients was on the symptomatic side, and 9 patients had codominance. Most patients (46/53) had varying degrees of bilateral TSS. The remaining 7 patients had TSS on the PT side and dysplasia of the transverse sinus on the asymptomatic side. Because all the patients had different degrees of unilateral/bilateral TSS, the CCS ranged from 1 to 4 in all the patients. Based on the CCS, the venous PT patients were divided into a high-degree TSS group (a score of 1–2) (*n* = 30) and a low-degree TSS group (a score of 3–4) (*n* = 23) (Table 1). MRV revealed no incidental TSS in the HCs (Figure 1).

### Cerebral Blood Flow Differences Among the Groups

The CBF values of patients with high-degree and low-degree TSS and HCs are exhibited in Table 2. Significant differences were found in the CBF value of the whole brain, GM and WM among the three groups (whole brain: *P* = 0.001; GM: *P* = 0.002; WM: *P* = 0.005). In the whole brain and GM, the patients with high-degree TSS showed significantly decreased CBF compared with the HCs (whole brain: 45.68 ± 5.58 ml/100 g/min vs. 51.58 ± 7.29 ml/100 g/min, *P* = 0.001; GM: 49.08 ± 6.26 ml/100 g/min vs. 55.03 ± 7.91 ml/100 g/min, *P* = 0.002); the patients with high-degree TSS exhibited significantly decreased CBF relative to compared with those with low-degree TSS (whole brain: 45.68 ± 5.58 ml/100 g/min vs. 50.30 ± 6.03 ml/100 g/min, *P* = 0.036; GM: 49.08 ± 6.26 ml/100 g/min vs. 54.04 ± 6.45 ml/100 g/min, *P* = 0.039); no significant difference in CBF was found in patients with low-degree TSS and HCs (*P* > 0.05). In the WM region, the patients with high-degree TSS exhibited significantly decreased CBF compared with that in the HCs (38.80 ± 3.90 ml/100 g/min vs. 42.83 ± 6.03 ml/100 g/min, *P* = 0.004).

**TABLE 2:** CBF measurement among the three groups.

	High-degree TSS	Low-degree TSS	HC	*P* ^a^
Whole brain CBF (ml/100 g/min)	45.68 ± 5.58	50.30 ± 6.03	51.58 ± 7.29	0.001
GM CBF (ml/100 g/min)	49.08 ± 6.26	54.04 ± 6.45	55.03 ± 7.91	0.002
WM CBF (ml/100 g/min)	38.80 ± 3.90	41.97 ± 4.62	42.83 ± 6.03	0.005

*The data are presented as mean ± standard deviation.*

*TSS, Transverse sinus stenosis; GM, gray matter; WM, white matter; HC, healthy control; CBF, cerebral blood flow.*

*^a^One-way analysis of variance.*

### White Matter Change

**FIGURE 2:**
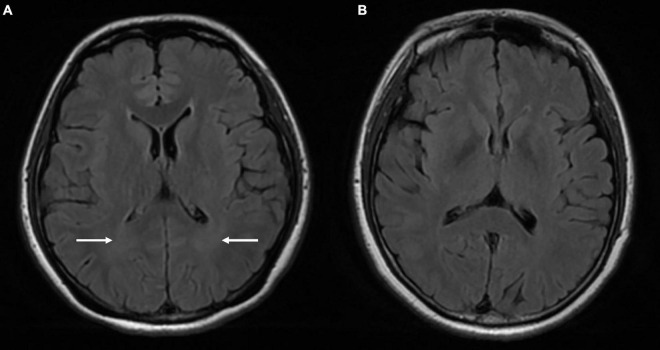
Comparison of the MRI findings between a 33-year-old female patient with TSS **(A)** and a 42-year-old female healthy control **(B)**. Venous PT patient with TSS: T2 FLAIR **(A)** shows symmetrical cloud-like WM hyperintensity in the bilateral periventricular area (white arrow) that was absent in the healthy control **(B)**. PT, pulsatile tinnitus; TSS, transverse sinus stenosis; FLAIR, fluid attenuated inversion recovery; WM, white matter.

Cloud-like WM hyperintensity on the T2 FLAIR sequence was observed in 17 of 30 patients with high-degree TSS (56.7%) and 13 of 23 patients with low-degree TSS (56.5%). The incidence of WM change was significantly higher in the above two patient groups than in the HC group (9 of 40; 22.5%; *P* < 0.05) (Figure 2).

## Discussion

In this study, we investigated brain perfusion and WM changes in venous PT patients with TSS. The results indicated that as the severity of TSS increased and overall venous drainage concomitantly decreased, the CBF values of the GM, WM and whole brain were significantly decreased. Additionally, the incidence of cloud-like WM hyperintensity was significantly higher in patients with TSS than in HCs. These findings confirm that TSS in venous PT patients may lead to pathological brain changes.

Venous PT is related to abnormal hemodynamics caused by different morphological changes (Li et al., 2021), including TSS, sigmoid sinus dehiscence with and without diverticulum, and jugular bulb abnormality (Dong et al., 2015; Kao et al., 2017; Hewes et al., 2020). TSS is one of the most common morphological abnormalities of venous PT and a cause of venous PT. Hewes et al. (2020) found that 84.6% of these patients had different degrees of bilateral TSS. After stent placement, the blood flow pattern became more regular (Han et al., 2017) and the PT disappeared immediately (Baomin et al., 2014). Therefore, TSS plays an essential role in the pathophysiology of venous PT.

Previous studies have reported decreased brain perfusion in patients with extracranial venous drainage disorder (Garaci et al., 2012; Zhou et al., 2019). Moreover, after stent placement or surgical restoration, brain hypoperfusion significantly improved or even disappeared (Zamboni et al., 2016; Zhou et al., 2019). Regarding pathophysiological mechanism, Zhou et al. (2019) believed that cerebral venous outflow disorder may contribute to increased intracranial venous pressure and that long-term venous hypertension reduces brain perfusion and disruption of the intracranial microvasculature. However, the above studies all used invasive examination including single-photon emission computed tomography (SPECT) and dynamic susceptibility contrast (DSC) MRI. Furthermore, these studies used semiquantitative or region-of-interest-based methods to assess CBF; these methods do not provide an accurate and comprehensive reflection of brain perfusion status. In this study, we used the ASL technique to non-invasively and quantitatively evaluate the CBF of the GM, WM and whole brain. Compared with HCs, patients with high-degree TSS showed significantly decreased CBF in the GM, WM and whole brain, demonstrating that TSS can lead to decreased brain perfusion. We also found that the patients with high-degree TSS showed significantly decreased CBF in whole brain and GM compared with the patients with low-degree TSS, and no significant difference was found between the patients with low-degree TSS and the HCs. This finding indicates that only a certain degree of TSS can lead to a significant decrease in brain perfusion. In contrast to the whole brain and GM, no significant difference was found in WM CBF between the patients with low-degree and high-degree TSS. Because the CBF of WM is significantly lower than that of GM under physiological conditions (Puig et al., 2020), we speculate that, in the two PT subgroups, the difference in WM CBF caused by TSS was too small to reach a statistical level.

Previous studies have mainly focused on the effects of aging and arterial diseases including cerebral small vessel disease, large vessel occlusion or stenosis on the WM (Lamar et al., 2010; Fennema-Notestine et al., 2016; Wang et al., 2016; Mistry et al., 2020). Chung et al. (2011) found that more severe age-related WM changes were observed in subjects with IJV reflux. This finding links WM changes with cerebral venous outflow disorder. Subsequently, their study further revealed that compared with patients with negative IJV reflux, the WM changes in Alzheimer’s disease (AD) patients with positive IJV reflux showed an increasing trend, indicating that cerebral venous outflow disorder may be involved in forming WM changes in AD patients (Chung et al., 2014). Zhou et al. (2019) found that 95.3% of patients with symptomatic IJV stenosis had bilateral symmetrical cloud-like WM hyperintensity. Bai et al. (2019) further found that cloud-like WM hyperintensity is a common feature of patients with IJV stenosis and patients with TSS. However, the above two studies did not include a control group for comparative analysis. In the present study, we found that the incidence of cloud-like WM hyperintensity is significantly higher in patients with TSS than in HCs. In contrast to the previous study, the participants in this study were relatively young (venous PT patients: 35.5 ± 7.0 years; HCs: 37.9 ± 6.7 years) and had no history of AD or arterial diseases. Currently, the underlying mechanism of the formation of cloud-like WM hyperintensity remains unclear. Zhou et al. (2019) believed that decreased brain perfusion was usually related to the formation of white matter lesions, particularly deep white matter lesions. We speculate that the relationships among decreased CBF, cloud-like WM hyperintensity, and TSS are as follows: TSS may inhibit the outflow of deoxyhemoglobin-rich venous blood and flow of hemoglobin-rich arterial blood into capillaries. These effects may affect the oxygen exchange between blood and brain tissues, resulting in decreased CBF using the ASL technique; long-term ischemia and hypoxia may lead to the demyelination of WM (Bai et al., 2019; Zhou et al., 2019).

In this study, we used the ASL technique, which uses arterial blood water as endogenous tracer (Binnewijzend et al., 2013), to non-invasively and quantitatively measure CBF. In contrast to traditional perfusion imaging techniques such as SPECT, dynamic contrast-enhanced MRI and DSC MRI, ASL can be used to perform repeated studies on subjects because of the lack of radioactivity and the non-use of exogenous contrast agents. This technique has been widely used in brain tumors, epilepsy, neurodegenerative and cerebrovascular diseases (Haller et al., 2016; Delgado et al., 2018; Barzgari et al., 2019) and provides valuable information for clinicians. Currently, the treatment principle for venous PT with TSS is based on eliminating PT without evaluating the brain perfusion and WM changes. Therefore, the decision to perform sigmoid sinus wall reconstruction or stent implantation does not depend on the presence or absence of brain change. In this study, however, decreased CBF and cloud-like WM hyperintensity in venous PT patients with TSS deserve more attention from clinicians. Appropriate and early intervention may help improve the neuropathological progress of patients.

This study has several limitations. First, because of the limited sample size, the effect of gender on CBF was not investigated. More venous PT patients will be included and grouped by gender. Second, previous studies have found that venous PT and idiopathic intracranial hypertension overlap in demography (Lansley et al., 2017; Hewes et al., 2020), that is, idiopathic intracranial hypertension may be present in venous PT patients. Idiopathic intracranial hypertension may affect brain perfusion (Bicakci et al., 2006). In this study, all the patients had no clinical symptoms of idiopathic intracranial hypertension; thus, lumbar puncture was not performed. Third, other comorbidities, such as diabetes and hypertension, can cause WM signal change (Fennema-Notestine et al., 2016). All the subjects in this study were young and middle-aged (patients: 35.5 ± 7.0 years; HCs: 37.9 ± 6.7 years), and none had systemic diseases such as hypertension, hyperlipidemia, and diabetes. Additionally, this is a cross-sectional study. We will enroll patients after the resolution of TSS and evaluate their neuroimaging characteristics to further verify the influence of TSS on the central nervous system.

## Conclusion

In summary, we found that TSS in venous PT patients can lead to decreased CBF and cloud-like WM hyperintensity. These representative neuroimaging findings may help understand the pathological TSS in venous PT.

## Data Availability Statement

The raw data supporting the conclusions of this article will be made available by the authors, without undue reservation.

## Ethics Statement

The studies involving human participants were reviewed and approved by the Ethics Committee of Beijing Friendship Hospital. The patients/participants provided their written informed consent to participate in this study.

## Author Contributions

XL, PZ, and ZW: conceptualization. XL and PZ: data curation and methodology. XL: formal analysis, investigation, visualization, software, and writing–original draft. RZ, JX, ZY, SG, and ZW: funding acquisition. HD, HL, XQ, XM, and CD: project administration. ZY, SG, and ZW: resources and supervision. ZW: validation. PZ and ZW: writing–review and editing. All authors contributed to the article and approved the submitted version.

## Conflict of Interest

The authors declare that the research was conducted in the absence of any commercial or financial relationships that could be construed as a potential conflict of interest. The reviewer SS has declared a shared parent affiliation with the authors at the time of the review.

## Publisher’s Note

All claims expressed in this article are solely those of the authors and do not necessarily represent those of their affiliated organizations, or those of the publisher, the editors and the reviewers. Any product that may be evaluated in this article, or claim that may be made by its manufacturer, is not guaranteed or endorsed by the publisher.

## References

[B1] Bae S. C. Kim D. K. Yeo S. W. Park S. Y. Park S. N. (2015). Single-center 10-year experience in treating patients with vascular tinnitus: diagnostic approaches and treatment outcomes. *Clin. Exp. Otorhinolaryngol.* 8 7–12. 10.3342/ceo.2015.8.1.7 25729489 PMC4338096

[B2] Bai C. Xu Y. Zhou D. Ding J. Yang Q. Ding Y. (2019). The comparative analysis of non-thrombotic internal jugular vein stenosis and cerebral venous sinus stenosis. *J. Thromb. Thrombolysis* 48 61–67. 10.1007/s11239-019-01820-1 30689154

[B3] Baomin L. Yongbing S. Xiangyu C. (2014). Angioplasty and stenting for intractable pulsatile tinnitus caused by dural venous sinus stenosis: a case series report. *Otol. Neurotol.* 35 366–370. 10.1097/MAO.0b013e3182990d52 24080976

[B4] Barzgari A. Sojkova J. Maritza Dowling N. Pozorski V. Okonkwo O. C. Starks E. J. (2019). Arterial spin labeling reveals relationships between resting cerebral perfusion and motor learning in Parkinson’s disease. *Brain Imaging Behav.* 13 577–587. 10.1007/s11682-018-9877-1 29744796 PMC6226370

[B5] Bicakci K. Bicakci S. Aksungur E. (2006). Perfusion and diffusion magnetic resonance imaging in idiopathic intracranial hypertension. *Acta Neurol. Scand.* 114 193–197. 10.1111/j.1600-0404.2006.00702.x 16911348

[B6] Binnewijzend M. A. Kuijer J. P. Benedictus M. R. van der Flier W. M. Wink A. M. Wattjes M. P. (2013). Cerebral blood flow measured with 3D pseudocontinuous arterial spin-labeling MR imaging in Alzheimer disease and mild cognitive impairment: a marker for disease severity. *Radiology* 267 221–230. 10.1148/radiol.12120928 23238159

[B7] Carvalho G. B. Matas S. L. Idagawa M. H. Tibana L. A. de Carvalho R. S. Silva M. L. (2017). A new index for the assessment of transverse sinus stenosis for diagnosing idiopathic intracranial hypertension. *J. Neurointerv. Surg.* 9 173–177. 10.1136/neurintsurg-2016-012605 27698231

[B8] Chung C. P. Beggs C. Wang P. N. Bergsland N. Shepherd S. Cheng C. Y. (2014). Jugular venous reflux and white matter abnormalities in Alzheimer’s disease: a pilot study. *J. Alzheimers Dis.* 39 601–609. 10.3233/JAD-131112 24217278

[B9] Chung C. P. Wang P. N. Wu Y. H. Tsao Y. C. Sheng W. Y. Lin K. N. (2011). More severe white matter changes in the elderly with jugular venous reflux. *Ann. Neurol.* 69 553–559. 10.1002/ana.22276 21391231

[B10] Delgado A. F. De Luca F. Hanagandi P. van Westen D. Delgado A. F. (2018). Arterial spin-labeling in children with brain tumor: a meta-analysis. *AJNR Am. J. Neuroradiol.* 39 1536–1542. 10.3174/ajnr.A5727 30072368 PMC7410530

[B11] Ding J. Guan J. Ji X. Meng R. (2019). Cerebral venous sinus stenosis may cause intracranial arterial hypoperfusion. *Clin. Neuroradiol.* 30 409–411. 10.1007/s00062-019-00833-w 31489451

[B12] Dong C. Zhao P. Yang J. Liu Z. Wang Z. (2015). Incidence of vascular anomalies and variants associated with unilateral venous pulsatile tinnitus in 242 patients based on dual-phase contrast-enhanced computed tomography. *Chin Med. J. (Engl.)* 128 581–585. 10.4103/0366-6999.151648 25698187 PMC4834766

[B13] Durst C. R. Ornan D. A. Reardon M. A. Mehndiratta P. Mukherjee S. Starke R. M. (2016). Prevalence of dural venous sinus stenosis and hypoplasia in a generalized population. *J. Neurointerv. Surg.* 8 1173–1177. 10.1136/neurintsurg-2015-012147 26747875

[B14] Eisenman D. J. Raghavan P. Hertzano R. Morales R. (2018). Evaluation and treatment of pulsatile tinnitus associated with sigmoid sinus wall anomalies. *Laryngoscope* 128 Suppl 2 S1–S13. 10.1002/lary.27218 29756346

[B15] Farb R. I. Vanek I. Scott J. N. Mikulis D. J. Willinsky R. A. Tomlinson G. (2003). Idiopathic intracranial hypertension: the prevalence and morphology of sinovenous stenosis. *Neurology* 60 1418–1424. 10.1212/01.wnl.0000066683.34093.e2 12743224

[B16] Favoni V. Pierangeli G. Cirillo L. Toni F. Abu-Rumeileh S. La Morgia C. (2019). Transverse sinus stenosis in refractory chronic headache patients: an observational study. *Front. Neurol.* 10:1287. 10.3389/fneur.2019.01287 31920914 PMC6921963

[B17] Fennema-Notestine C. McEvoy L. K. Notestine R. Panizzon M. S. Yau W. W. Franz C. E. (2016). White matter disease in midlife is heritable, related to hypertension, and shares some genetic influence with systolic blood pressure. *Neuroimage Clin.* 12 737–745. 10.1016/j.nicl.2016.10.001 27790395 PMC5071546

[B18] Garaci F. G. Marziali S. Meschini A. Fornari M. Rossi S. Melis M. (2012). Brain hemodynamic changes associated with chronic cerebrospinal venous insufficiency are not specific to multiple sclerosis and do not increase its severity. *Radiology* 265 233–239. 10.1148/radiol.12112245 22915599

[B19] Guo X. Zhu J. Zhang N. Zhang L. Qi Y. Cai H. (2018). Altered neurovascular coupling in neuromyelitis optica. *Hum. Brain Mapp.* 40 976–986. 10.1002/hbm.24426 30315685 PMC6865682

[B20] Ha J. Y. Choi Y. H. Lee S. Cho Y. J. Cheon J. E. Kim I. O. (2019). Arterial spin labeling MRI for quantitative assessment of cerebral perfusion before and after cerebral revascularization in children with moyamoya disease. *Korean J. Radiol.* 20 985–996. 10.3348/kjr.2018.0651 31132824 PMC6536794

[B21] Haller S. Zaharchuk G. Thomas D. L. Lovblad K. O. Barkhof F. Golay X. (2016). Arterial spin labeling perfusion of the brain: emerging clinical applications. *Radiology* 281 337–356. 10.1148/radiol.2016150789 27755938

[B22] Han Y. Yang Q. Yang Z. Xia J. Su T. Yu J. (2017). Computational fluid dynamics simulation of hemodynamic alterations in sigmoid sinus diverticulum and ipsilateral upstream sinus stenosis after stent implantation in patients with pulsatile tinnitus. *World Neurosurg.* 106 308–314. 10.1016/j.wneu.2017.06.168 28698087

[B23] Hewes D. Morales R. Raghavan P. Eisenman D. J. (2020). Pattern and severity of transverse sinus stenosis in patients with pulsatile tinnitus associated with sigmoid sinus wall anomalies. *Laryngoscope* 130 1028–1033. 10.1002/lary.28168 31301193

[B24] Hofmann E. Behr R. Neumann-Haefelin T. Schwager K. (2013). Pulsatile tinnitus: imaging and differential diagnosis. *Deutsch. Arztebl. Int.* 110 451–458. 10.3238/arztebl.2013.0451 23885280 PMC3719451

[B25] Kao E. Kefayati S. Amans M. R. Faraji F. Ballweber M. Halbach V. (2017). Flow patterns in the jugular veins of pulsatile tinnitus patients. *J. Biomech.* 52 61–67. 10.1016/j.jbiomech.2016.12.008 28057349 PMC5415495

[B26] Lamar M. Charlton R. A. Morris R. G. Markus H. S. (2010). The impact of subcortical white matter disease on mood in euthymic older adults: a diffusion tensor imaging study. *Am. J. Geriatr. Psychiatry* 18 634–642. 10.1097/JGP.0b013e3181cabad1 20220594

[B27] Lansley J. A. Tucker W. Eriksen M. R. Riordan-Eva P. Connor S. E. J. (2017). Sigmoid Sinus diverticulum, dehiscence, and venous sinus stenosis: potential causes of pulsatile tinnitus in patients with idiopathic intracranial hypertension? *AJNR Am. J. Neuroradiol.* 38 1783–1788. 10.3174/ajnr.a5277 28705815 PMC7963710

[B28] Li X. Qiu X. Ding H. Lv H. Zhao P. Yang Z. (2021). Effects of different morphologic abnormalities on hemodynamics in patients with venous pulsatile tinnitus: a four-dimensional flow magnetic resonance imaging study. *J. Magn. Reson. Imaging* 53 1744–1751. 10.1002/jmri.27503 33491233 PMC8248416

[B29] Lyu A. R. Park S. J. Kim D. Lee H. Y. Park Y. H. (2018). Radiologic features of vascular pulsatile tinnitus - suggestion of optimal diagnostic image workup modalities. *Acta Otolaryngol.* 138 128–134. 10.1080/00016489.2017.1385847 28990828

[B30] Mistry E. A. Mistry A. M. Mehta T. Arora N. Starosciak A. K. La Rosa F. (2020). White matter disease and outcomes of mechanical thrombectomy for acute ischemic stroke. *AJNR Am. J. Neuroradiol.* 41 639–644. 10.3174/ajnr.A6478 32165366 PMC7144640

[B31] Morris P. P. Black D. F. Port J. Campeau N. (2017). Transverse sinus stenosis is the most sensitive MR imaging correlate of idiopathic intracranial hypertension. *AJNR Am. J. Neuroradiol.* 38 471–477. 10.3174/ajnr.a5055 28104635 PMC7959992

[B32] Puig O. Henriksen O. M. Vestergaard M. B. Hansen A. E. Andersen F. L. Ladefoged C. N. (2020). Comparison of simultaneous arterial spin labeling MRI and (15)O-H2O PET measurements of regional cerebral blood flow in rest and altered perfusion states. *J. Cereb. Blood Flow Metab.* 40 1621–1633. 10.1177/0271678X19874643 31500521 PMC7370368

[B33] Saindane A. M. Bruce B. B. Desai N. K. Roller L. A. Newman N. J. Biousse V. (2014). Transverse sinus stenosis in adult patients with Chiari malformation type I. *AJR Am. J. Roentgenol.* 203 890–896. 10.2214/AJR.14.12528 25247957 PMC4277891

[B34] Scott J. N. Farb R. I. (2003). Imaging and anatomy of the normal intracranial venous system. *Neuroimaging Clin. N. Am.* 13 1–12. 10.1016/s1052-5149(02)00062-x 12802937

[B35] Wang G. P. Zeng R. Liu Z. H. Liang X. H. Xian J. F. Wang Z. C. (2014). Clinical characteristics of pulsatile tinnitus caused by sigmoid sinus diverticulum and wall dehiscence: a study of 54 patients. *Acta Otolaryngol.* 134 7–13. 10.3109/00016489.2013.831479 24032538

[B36] Wang M. Norman J. E. Srinivasan V. J. Rutledge J. C. (2016). Metabolic, inflammatory, and microvascular determinants of white matter disease and cognitive decline. *Am. J. Neurodegener. Dis.* 5 171–177. 28078193 PMC5218857

[B37] Werner J. D. Siskin G. P. Mandato K. Englander M. Herr A. (2011). Review of venous anatomy for venographic interpretation in chronic cerebrospinal venous insufficiency. *J. Vasc. Interv. Radiol.* 22 1681–1690. 10.1016/j.jvir.2011.08.018 21975259

[B38] Zamboni P. Menegatti E. Cittanti C. Sisini F. Gianesini S. Salvi F. (2016). Fixing the jugular flow reduces ventricle volume and improves brain perfusion. *J. Vasc. Surg. Venous Lymphat. Disord.* 4 434–445. 10.1016/j.jvsv.2016.06.006 27638998

[B39] Zhao P. Lv H. Dong C. Niu Y. Xian J. Wang Z. (2016). CT evaluation of sigmoid plate dehiscence causing pulsatile tinnitus. *Eur. Radiol.* 26 9–14. 10.1007/s00330-015-3827-8 25991486

[B40] Zhou D. Ding J. Asmaro K. Pan L. Ya J. Yang Q. (2019). Clinical characteristics and neuroimaging findings in internal jugular venous outflow disturbance. *Thromb. Haemost.* 119 308–318. 10.1055/s-0038-1676815 30605919

[B41] Zhuo C. Zhu J. Qin W. Qu H. Ma X. Yu C. (2017). Cerebral blood flow alterations specific to auditory verbal hallucinations in schizophrenia. *Br. J. Psychiatry* 210 209–215. 10.1192/bjp.bp.115.174961 28104737

