# Supplementary material for: Transverse Sinus Stenosis in Venous Pulsatile Tinnitus Patients May Lead to Brain Perfusion and White Matter Changes
Source: Front Neurosci. 2021 Dec 8;15:732113. doi: 10.3389/fnins.2021.732113 (PMC8694213; doi:10.3389/fnins.2021.732113)
Supplement: Supplementary file 1 [file Image_1.pdf]

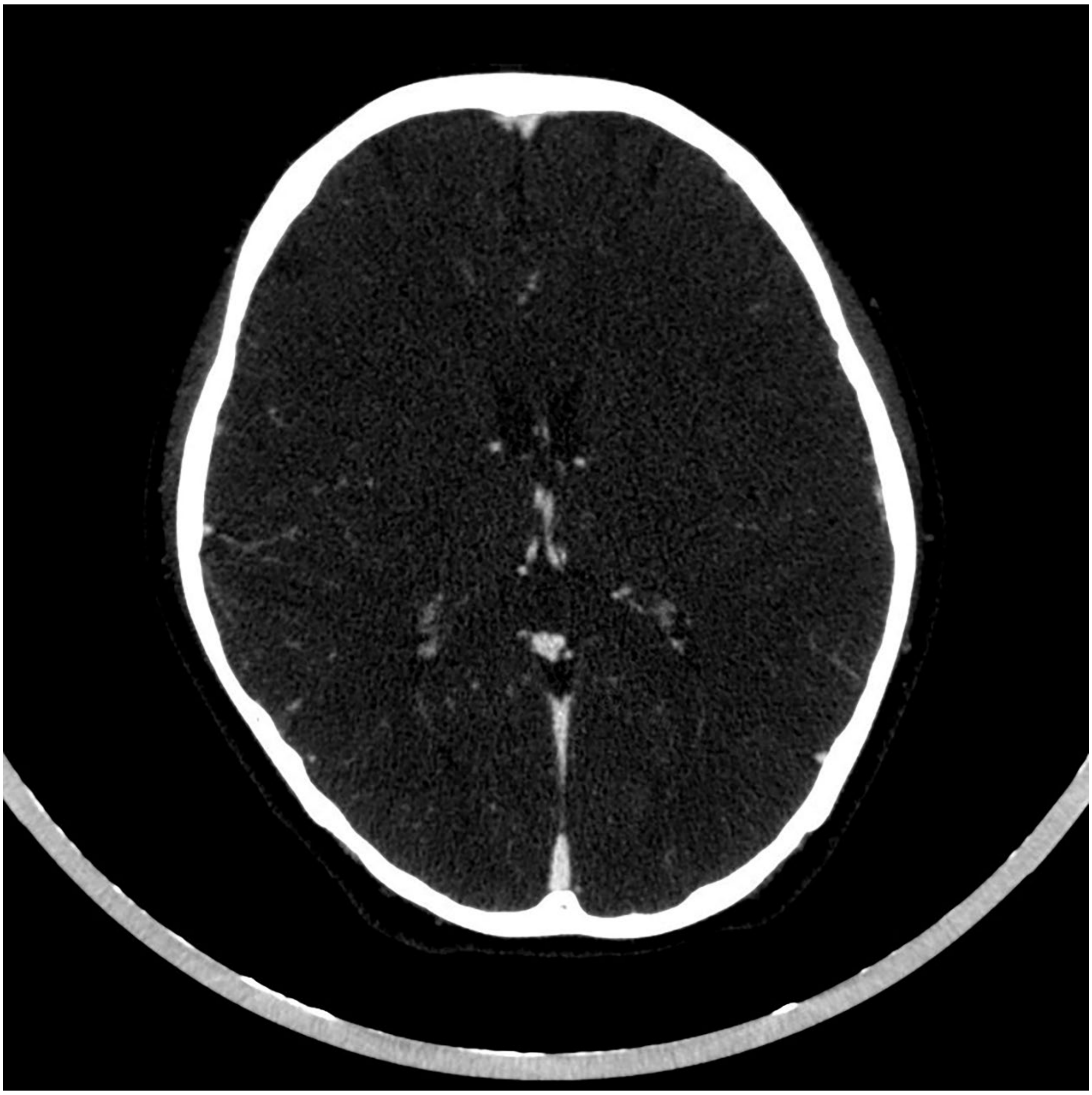

Image 1 CTV showed the distal superior sagittal sinus in a venous PT patient. PT: pulsatile tinnitus
